# Testing the mindfulness-to-meaning theory: Evidence for mindful positive emotion regulation from a reanalysis of longitudinal data

**DOI:** 10.1371/journal.pone.0187727

**Published:** 2017-12-06

**Authors:** Eric L. Garland, Adam W. Hanley, Phillipe R. Goldin, James J. Gross

**Affiliations:** 1 College of Social Work, University of Utah, Salt Lake City, Utah, United States of America; 2 Center on Mindfulness and Integrative Health Intervention Development, Salt Lake City, Utah, United States of America; 3 Huntsman Cancer Institute, University of Utah, Salt Lake City, Utah, United States of America; 4 Betty Irene Moore School of Nursing, University of California – Davis, Sacramento, California, United States of America; 5 Stanford University, Stanford, California, United States of America; Bangor University, UNITED KINGDOM

## Abstract

**Background and objective:**

The Mindfulness to Meaning Theory (MMT) provides a detailed process model of mindful positive emotion regulation.

**Design:**

We conducted a post-hoc reanalysis of longitudinal data (N = 107) derived from a RCT of mindfulness-based stress reduction (MBSR) versus cognitive-behavioral therapy (CBT) for social anxiety disorder to model the core constructs of the MMT (attentional control, decentering, broadened awareness, reappraisal, and positive affect) in a multivariate path analysis.

**Results:**

Findings indicated that increases in attentional control from baseline to post-training predicted increases in decentering by 3 months post-treatment (p<.01) that in turn predicted increases in broadened awareness of interoceptive and exteroceptive data by 6 months post-treatment (p<.001). In turn, broadened awareness predicted increases in the use of reappraisal by 9 months post-treatment (p<.01), which culminated in greater positive affect at 12 months post-treatment (p<.001). MBSR led to significantly greater increases in decentering (p<.05) and broadened awareness than CBT (p<.05). Significant indirect effects indicated that increases in decentering mediated the effect of mindfulness training on broadening awareness, which in turn mediated enhanced reappraisal efficacy.

**Conclusion:**

Results suggest that the mechanisms of change identified by the MMT form an iterative chain that promotes long-term increases in positive affectivity. Though these mechanisms may reflect common therapeutic factors that cut across mindfulness-based and cognitive-behavioral interventions, MBSR specifically boosts the MMT cycle by producing significantly greater increases in decentering and broadened awareness than CBT, providing support for the foundational assumption in the MMT that mindfulness training may be a key means of stimulating downstream positive psychological processes.

## Introduction

Cognitive, emotional, and physical health benefits have been linked to mindfulness, or the metacognitive tendency to intentionally attend to the flow of experience with equanimity [1–4]. Mindfulness is believed to be a natural psychological capacity [5] capable of being enhanced by intentional practice [6], such as mindful breathing meditations delivered within mindfulness-based interventions (MBIs)–interventions which have been shown in meta-analyses to improve mental health [7] and physical functioning [8] The formal practice of mindfulness meditation involves repeated placement of attention onto an object while alternately acknowledging and letting go of distracting thoughts and emotions. Objects of mindfulness practice can include the sensation of breathing; the sensation of walking; interoceptive and proprioceptive feedback about the body’s internal state, movement, and position; visual stimuli such a candle flame or running water; mental contents such as thoughts or feelings; or the quality of awareness itself. Such practices have been shown to increase the disposition to be mindful in everyday life [9], and to produce changes in neurocognitive function consistent with increased attentional control, emotion regulation, and self-awareness [10].

At the same time, recent evidence demonstrates that activities other than meditation and forms of clinical intervention other than MBIs can increase mindfulness. For instance, involvement with cognitive behavioral therapy [11] and even washing dishes in an intentional manner as an informal meditation practice [12] have been shown to increase mindfulness. As such, a wide variety of pursuits may yield substantive changes in mindfulness, and thereby confer downstream benefits of mindfulness on psychological and physical health. Given the benefits of mindfulness and the diverse means by which it may be cultivated, mindfulness is positioned as a unique construct in the service of promoting well-being.

In the pursuit of alleviating suffering, considerable empirical and theoretical efforts have been made to clarify the mechanisms by which mindfulness reduces unpleasant cognitive, emotional, and physical experiences [13–15]. However, considerably less effort has been directed towards specifying the mechanisms by which mindfulness encourages positive experiences and psychological well-being. This is an important oversight with clinical relevance, given linkages between positive emotional processes and health [16,17]. Indeed, positive affect stimulates the neuroendocrine, autonomic, and immune systems in salutary ways that are independent of negative affect [18], promote pain relief [19], and engender physical and psychological benefits in part by enhancing higher-order cognitive attitudes and processes like optimism [20], reappraisal [21], and meaningfulness in life [22]. In turn, indices of eudaimonic well-being, like purpose in life, have been shown to predict improved function in physiological systems involved in the stress response [23] and are linked with a genomic profile that is potentially health-generating [24].

Recently, the Mindfulness to Meaning Theory (MMT) [25] was proposed as a model of mindful positive emotion regulation to fill the lacuna of formalized theory connecting mindfulness to more enduring, positive markers of health, such as eudaimonic well-being. The MMT provides a detailed process model explicating changes in downstream perceptual tendencies as well as emotion regulation strategies proposed to emerge from the state of mindfulness. The MMT asserts that 1) engaging attentional control in the face of stress fosters 2) decentering from stress appraisals into a metacognitive state, which yields a 3) broadening of awareness to encompass previously unattended interoceptive and exteroceptive sensory information. This novel contextual information is then 4) processed and integrated into new adaptive reappraisals of self and world, ultimately 5) resulting in a durable form of positive affectivity and the sense of meaningfulness in life. Though the MMT was originally developed to account for the ways in which mindfulness training (such as that afforded by MBIs) might promote positive emotion regulation, the MMT does not specify mindfulness meditation per se but instead specifies mechanisms implicated in the state and trait of mindfulness (e.g., attentional control, decentering). Therefore, the MMT may delineate transtherapeutic processes linking mindfulness to reappraisal and positive affect that arise as a result of any psychological intervention capable of stimulating the state and trait of mindfulness. For a full description of the MMT, see Garland and colleagues [26,27].

### Key processes in the MMT linking mindfulness to reappraisal

Attentional control, or the ability to sustain attention on an object in the context of distraction and deliberately shift (i.e., re-orient) attentional focus [28], is a fundamental mechanism of mindfulness. Dispositional mindfulness, a psychological propensity strengthened by mindfulness training [9], is positively associated with sustained attention [29,30] and the ability to re-orient attention in the face of emotional stimuli [31]. Many MBIs seek to promote both attentional capacities (i.e., focusing and shifting) through direct instruction on focused-attention practices (i.e., attending to an identified object such as the breath) as well as open-monitoring practices (i.e., attending indiscriminately to the flux of experience) [32]. While evidence is not conclusive [14] empirical research demonstrates that mindfulness training supports sustained attention capacity, generally in advanced meditators, [33,34] and augments attentional orienting capacity in the early stages of meditation [35,36]–this latter finding has been paralleled by evidence of the effects of MBIs on reducing attentional bias towards emotionally threatening cues [37,38]. As a result of such enhanced attentional capacity, mindfulness practitioners (and individuals who experience increased dispositional mindfulness through CBT and other interventions) may be better able to regulate their attention in response to distressing thoughts and emotions. Better attentional regulation may promote decentering from difficult psychological content as attentional resources can be more intentionally mobilized to initiate cognitive coping strategies.

Decentering, the act of disengaging from sensory, cognitive, or emotional phenomenon to achieve a psychological or reflective distance in relation to internal experiences [39], is held to be an essential mechanism of mindfulness by some theorists [3,40]. Other theorists suggest that while decentering shares considerable conceptual overlap with mindfulness, decentering is a distinct construct from mindfulness [40,41] that can also be stimulated by CBT [39]. In the MMT, decentering is believed to clear working memory of stress appraisals, undo attentional biases associated with stimulus-contingent, maladaptive cognitive schemas, and disrupt automatic behavioral repertoires. The MMT proposes that through decentering, attention is disengaged from habitual cognitive sets and broadened into a state of metacognitive awareness, a mode of apperception in which one monitors the object of cognition as well as the meta-level of awareness in which dynamic models (e.g., schemas) of the object level are contained (i.e., an awareness of the quality of awareness itself) [42]. In other words, the MMT operationalizes decentering as the *process* by which the *state* of metacognitive awareness emerges, a state in which both the attentional object and the field in which the object is perceived may exist in awareness simultaneously. Recent conceptual models and related empirical evidence indicate that decentering is linked with metacognitive awareness, as well as reduced reactivity to and disidentification from thoughts [40]. Although models differ concerning whether decentering or metacognitive awareness is taken as primary [25,40], the relationship between the constructs is commonly viewed as recursive. Moreover, contemplative science theories posit that decentering is an initial stage in the existential progression towards deepening metacognitive awareness of the field in which subject and object is construed–a form of metacognitive self-regulation that results in insight into the ‘intentionality of concepts’ and thereby enhances the fluidity of conceptual processing [43]. Regardless of its relationship with decentering, metacognitive awareness has been long held as a mechanism of mindfulness [4,44]. Hence, the practice of mindfulness could be characterized as repeated instances of decentering from emotional events and/or mental proliferation into a metacognitive state. Indeed, mindfulness training has been associated with increased decentering and metacognitive awareness [44–46].

In the MMT, the construct of broadened awareness of interoceptive and exteroceptive information refers to increased access to perceptions of the internal milieu and the external environment made possible by decentering into a metacognitive state–yielding contextual information that was previously constrained by the narrowed attentional perspective induced by stress and negative affective states. This expansion of the field of awareness is theorized to facilitate reconfiguration of appraisals by integrating previously unattended, positive contextual features into apperception of neutral and negative events, resulting in a more balanced set of interoceptive and exteroceptive information from which reappraisals can be generated. In this way, broadened awareness of internal and external context is theorized in the MMT to be instrumental in positive reappraisal, or the process through which stressful events are reconstrued as benign, meaningful, or growth promoting [47]. In support of this contention, heightened levels of interoceptive awareness enhance electrophysiological and subjective markers of reappraisal efficacy [48] and are associated with increased use of reappraisal [49]. Similarly, attention shifting is linked with reappraisal efficacy [50] and increased attention to positive information has been associated with the propensity to reconstrue adversity as a source of personal growth [51]–a propensity that has been shown to be enhanced by mindfulness [52]. Hence, broadening awareness to encompass and process a larger array of contextual information may provide the novel input necessary to construct a reappraisal narrative. For evidence of a similar assertion see Wadlinger and Isaacowitz [53]. In turn, experimental evidence indicates that positively reappraising negative events enhances positive affectivity and psychological well-being [21,54,55]

Unifying these conceptual and empirical considerations, the MMT proposes the *mindful reappraisal hypothesis* [27], which states that mindful decentering promotes reappraisal by broadening awareness, thereby increasing access to previously unattended contextual data from which new appraisals can be constructed. In turn, reappraisal is identified within the MMT as a primary, cognitive self-regulatory mechanism that may engender positive emotions and ultimately introduce greater flexibility in the construction of meaning from experience. Given relations between positive affect and health, the MMT may hold considerable utility for the theory and practice of psychotherapeutic intervention.

### The direct relation between mindfulness and reappraisal

Mounting evidence supports the mindful reappraisal hypothesis indirectly by establishing bivariate associations between these core constructs within the MMT’s theoretical framework [13] (for additional reviews establishing relations between attentional control, decentering, and broadened awareness, see [40]. The proposed relation between mindfulness and reappraisal has also been supported by recent empirical work indicating a direct relation between these two constructs [24]. It is particularly important to establish the direct relationship between mindfulness and reappraisal as the conceptual nature of reappraisal is sometimes posed to be antithetical to the ostensibly non-conceptual state of mindfulness [56]. However, better understanding the exchange between the non-conceptual mechanisms implicated in mindfulness and the conceptual field of day-to-day life holds considerable clinical and theoretical utility. Though the prospect of suspending conceptual processing for extended periods in daily life is purportedly achievable for long-term meditators, re-engaging conceptual thought (i.e., appraisals) in the immediate wake of mindfulness remains a necessity for novice mindfulness practitioners. Evidence from a number of correlational [57], quasi-experimental [58,59], and experimental studies [11,60,61] suggests that mindfulness may support reappraisal. In contrast to CBT which largely focuses on modifying propositional (i.e., declarative, semantic) meanings, mindfulness may bolster reappraisal by modifying implicational meanings relevant to the stressor context and one’s broader sense of self [62]. Implicational meaning goes beyond the explicit, conceptual framing of an experience to a felt, holistic interpretation of the experience, a type of meaning-making that has been theorized to be an especially potent means of transforming one’s experience of affective distress [63]. Accessing metacognitive insight through mindful decentering has been posited as a means of facilitating the remapping of implicational meanings [43]–which hypothetically would result in contextual reappraisals. Furthermore, the relationship between mindfulness and positive reappraisal may operate in an cross-lagged fashion such that increases in state mindfulness across time promote increases in positive reappraisal, a finding recently observed in temporally-dynamic causal modeling attempts [64].

### The present study

Despite its theoretical coherence, empirical support for the MMT has been “patch-worked” together by demonstrating bivariate relationships between the core model components in separate studies. Only one prior study has simultaneously examined linkages between multiple core constructs specified in the MMT in a multivariate path analysis of cross-sectional data obtained from a sample of cancer survivors [65], but this analysis was limited in its ability to ascertain time-ordering of these constructs and their responsivity to intervention. To date, no comprehensive examination of the MMT has been conducted with longitudinal data. The next step in testing and refining the MMT is to situate the identified, core model components together in a single longitudinal analysis, investigating the entire theoretical model in response to clinical intervention. To that end, in this post-hoc secondary data analysis, the MMT was modeled with data from a RCT of participants with social anxiety disorder (SAD) who were randomized to either 12 weeks of a mindfulness-based stress reduction (MBSR) course or cognitive-behavioral therapy (CBT) delivered in a group format. Though this trial was designed to examine the differential efficacy and mechanisms of MBSR and CBT for social anxiety, a number of the mediating variables collected in this study map onto the MMT. We employed the longitudinal dataset from this trial to conduct post-hoc modeling of the MMT with the hope that the mechanistic insights gained from this secondary analysis might have broader application.

In the Goldin et al. trial [11], relative to a wait-list control, both CBT and MBSR significantly improved anxiety while increasing mindfulness and reappraisal, and increases in mindfulness and reappraisal did not significantly differ between these two active interventions. The finding that MBSR significantly increases reappraisal (without providing explicit reappraisal training) provides the strongest and most direct support for the mindful reappraisal hypothesis of MMT yet. Though pedagogical and experiential techniques in CBT and MBSR may be substantively different, both treatment approaches appear to operate on common factors via underlying transtherapeutic change mechanisms. Yet, mindfulness training through MBSR may specifically stimulate the MMT process by selectively targeting core MMT constructs integral to the practice of mindfulness (e.g., decentering, broadening of awareness).

To model the change process in accordance with the MMT, we hypothesized that increases in attentional control [from Time 1 (baseline) to Time 2 (immediately post-treatment)] would predict increases in decentering (by Time 3; 3 months post-treatment), that would in turn predict increases in broadened awareness of interoceptive and exteroceptive data (by Time 4; 6 months post-treatment). In turn, broadened would predict increased use of reappraisal (by Time 5; 9 months post-treatment), which would culminate in greater positive affect (by Time 6; 12 months post-treatment). In addition to testing these hypothesized linkages between these core constructs as specified by the MMT, we also tested the influence of MBSR vs CBT on therapeutic change in these constructs.

## Materials and method

### Participants and procedures

Participants (*n* = 107) were included in this study if they met criteria for a principal diagnosis of social anxiety disorder. Exclusion criteria included: involvement with psychotherapy or pharmacotherapy during the previous year; participation in CBT for an anxiety disorder during the previous two years; a history of mindfulness practice involvement either through an MBSR course, long-term meditation retreat, or individual practice, and history or current neurological disorder, cardiovascular disorder, thought disorder, bipolar disorder, or substance use disorder.

Participants were randomized into one of three, 12-week conditions: 1) CBT, 2) MBSR, or 3) wait-list control. Following the waiting period, participants in the wait-list were randomly assigned and crossed over into either CBT or MBSR. To maximize the effective sample size in the present secondary data analysis, we examined data from all participants following randomization to CBT or MBSR (including those participants who were originally randomized to the wait-list condition). Pretreatment assessments, measuring attentional control, dispositional mindfulness, emotion regulation and positive affect were also administered at posttreatment and at 12 months after posttreatment. An abbreviated assessment battery, including measures of dispositional mindfulness and emotion regulation, were also completed at three months, six months and nine months posttreatment. Treatment was provided at no cost to participants and they were paid $150 dollars to complete the follow-up assessments. Participants provided written informed consent and Stanford University IRB approved this study.

Participant demographics are depicted in Table 1. Further details pertaining to participant characteristics, recruitment, screening, and retention are reported in Goldin et al. [11].

**Table 1:** Participant demographics (N = 107).

Measure	
Female, N (%)	59 (55%)
Age, (*SD)*	32.84 (8.13)
Race, N (%)	
American Indian/Alaskan Native	1 (1%)
Asian	39 (36%)
African American	1 (1%)
Caucasian	49 (46%)
Latino	9 (8%)
Multiracial	8 (8%)
Income level, N (%)	
Under $10,000	7 (8%)
$10–25,000	8 (10%)
$25–50,000	16 (19%)
$50–75,000	12 (15%)
$75–100,000	12 (15%)
Over $100,000	28 (34%)
Years of Education, (*SD)*	16.53 (2.46)
Marital Status, N (%)	
Single	60 (57%)
Married	34 (32%)
Living with Partner	10 (10%)
Divorced	1 (1%)
Other	1 (1%)

### Interventions

#### MBSR

MBSR followed the standard curriculum [66] with the exception that instead of holding a 1-day meditation retreat, participants had four additional weekly group sessions between the standard Class 6 and to create 12 weekly 2.5 hour sessions to match the CBT protocol in duration and time. MBSR involves mindful breathing, body scan, informal mindfulness, and lovingkindness meditation practices.

#### CBT

CBT followed a standardized CBT group therapy protocol [67] and was delivered over 12 sessions of 2.5 hours each. Treatment involved psychoeducation, cognitive restructuring skills, graduated exposure to feared social situations, and relapse prevention.

### Measures

#### Attentional control

The Attentional Control Scale (ACS; *α* = .85 for focusing and *α* = .74 for shifting subscales in this sample) is a 19-item measure scored on a 7-point Likert scale (1 = “strongly disagree” to 7 = “strongly agree”) that assesses respondents’ abilities to both focus (“When concentrating I ignore feelings of hunger or thirst”) as well as shift (“I can quickly switch from one task to another”) attention [68].

#### Decentering

The Five Facet Mindfulness Questionnaire’s (FFMQ) non-reactivity subscale (*α* = .72 in this sample), is a 7-item scale measure on a 5-point Likert Scale (1 = “never or very rarely true” to 5 = “very often or always true”) was used to measure decentering [69]. The non-reactivity subscale includes items tapping key features of decentering including disidentification (e.g., “I watch my feelings without getting lost in them”) and reduced reactivity (e.g., “When I have distressing thoughts or images I am able to just notice them without reaction.”

#### Broadened awareness of interoceptive and exteroceptive data

The FFMQ observing subscale (*α* = .75 in this sample), is an 8-item measure scored on a 5-point Likert Scale (1 = “never or very rarely true” to 5 = “very often or always true”) that assesses respondents’ tendencies to become aware of internal and external experiences [69], including pleasant and neutral perceptions and body sensations [70]. The FFMQ observing subscale reflects a broadening of awareness to encompass usually unattended neutral (e.g., sensations of bodily movement, background sounds) or pleasant stimuli (e.g., the sun on one’s face, the breeze through one’s hair). Such an expanded attentional capacity is theoretically consistent with the MMT's conceptualization of attentional broadening as the part of the mindful self-regulation process in which the practitioner's awareness, previously constricted by the stress response, expands to include neutral and positive elements of the environment that had gone previously unnoticed. In that regard, higher scores on the observe facet are significantly correlated with increased attentional alerting to novel stimuli [71], supporting our use of this measure to tap broadened awareness of previously unattended data.

#### Reappraisal

The Emotion Regulation Questionnaire’s (ERQ; *α* = .93 in this sample) reappraisal self-efficacy subscale [54], is a 6-item measure scored on a 7-point Likert scale (1 = “strongly disagree” to 7 = “strongly agree”) that assesses one’s self-reported ability to regulate emotion by reconstruing the meaning of adverse situations (e.g., “When I really want to, I am very capable of changing the way I'm thinking about a situation when I want to feel less negative emotion”).

#### Positive affect

The Positive Affect and Negative Affect Scale’s (PANAS; *α* = .81 in this sample) positive affect subscale is a 10-item measure scored on a 5-point Likert scale (1 = “very slightly or not at all” to 5 = “extremely”) that assesses positive affect using a variety of adjectives characteristic of positive emotions.

### Statistical analysis

Multivariate path analysis was used to model the core constructs in the MMT theory: attentional control, decentering, broadened awareness, reappraisal, and positive affect. Each variable in the model was regressed on its pretreatment score, to reflect a residualized change score. To represent the autoregressive nature of the data more accurately, each successive data point was modeled as its own observed variable with its own error term to account for measurement error. Variables in the model were organized in a temporally progressive fashion according to the MMT such that attentional control was measured at posttreatment, decentering at three months after posttreatment, broadened awareness at six months after posttreatment, reappraisal at nine after months posttreatment and positive affect at 12 months after posttreatment. We chose to create residualized change scores with pretreatment levels to account for the learning and cumulative change that occurred within treatment as well as in the follow-up period for those variables that were measured at later time points. Though we could have created change scores that only reflected successive change (e.g., T2 to T3, T3 to T4, etc.), this approach would have not taken into account the influence of psychological development that may have occurred during and after the MBSR and CBT interventions, which we thought would be critical to the process described by the MMT. Finally, treatment group membership was represented as an exogenous variable in the model, with effects of treatment modeled via paths the from the treatment group indicator to the variables representing residualized change in attentional control, decentering, broadened awareness, reappraisal, and positive affect.

Because our hypothetical model, like all causal models, is prone to specification error, other alternative models were assessed to ensure that significant path coefficients identified were not artifactual. To that end, alternative model configurations were examined in which we replaced each variable at each time point in an exhaustive fashion, to test whether variable combinations and linkages that were not specified in the MMT fit the data better.

## Results

### Multivariate path analysis

**Fig 1:**
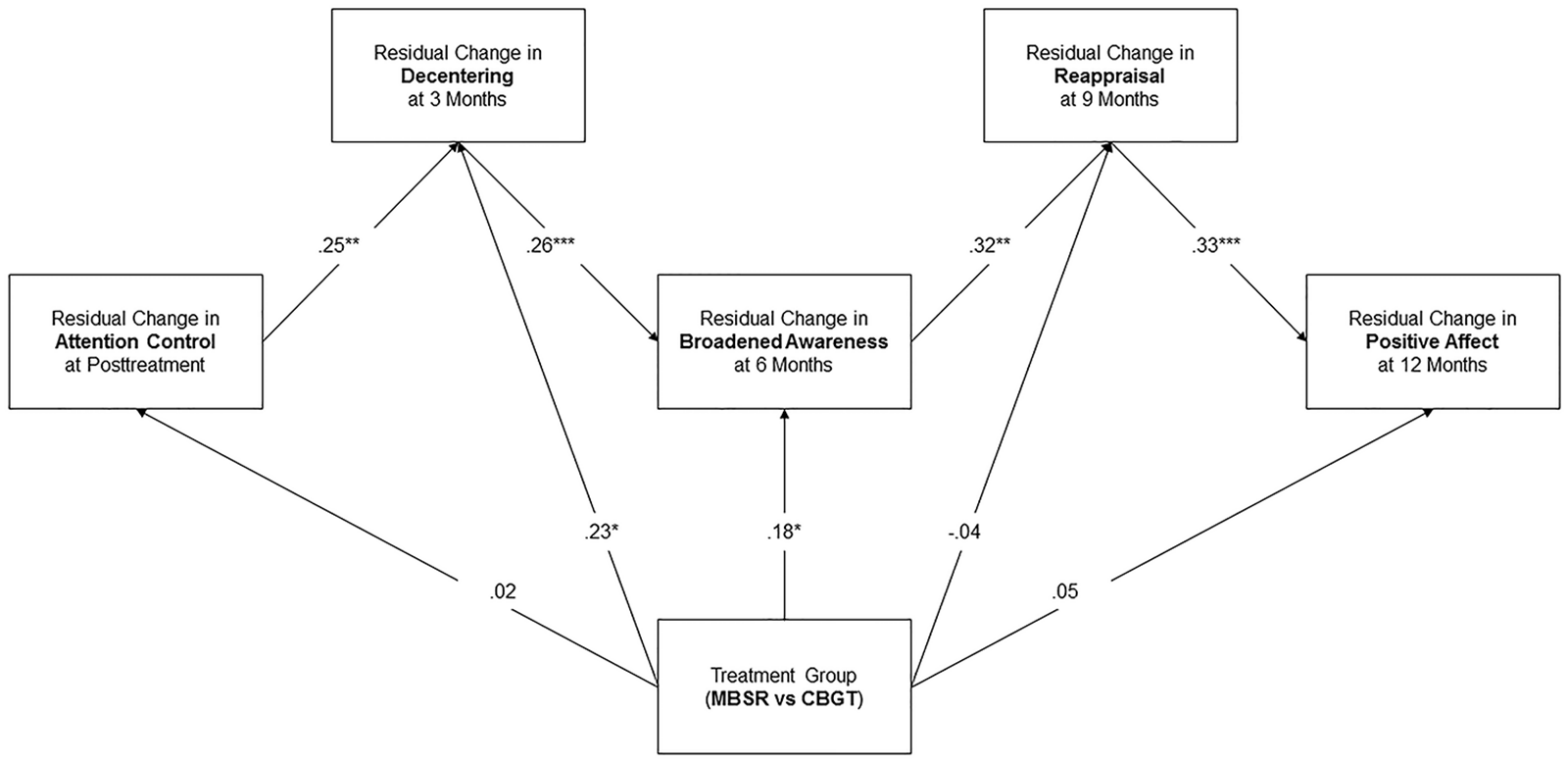
Final multivariate path model of the mindfulness-to-meaning theory. Note: Change was computed in residualized change scores (follow-up levels adjusted for pre-treatment levels). All paths are statistically significant. Model fit was excellent, χ^2^/*df* = 1.17, *p* = .22, CFI = .97, RMSEA = .04 (.00, .08).

In the multivariate path model, all paths in the MMT model were significant (Fig 1) and model fit was excellent (χ^2^/*df* = 1.17, *p* = .22, CFI = .97, RMSEA = .04 (.00, .08)). With respect to treatment group differences, we observed significant direct effects from the variable representing treatment condition to decentering and broadened awareness: MBSR was associated with significantly greater increases in decentering by Time 3 (B = 1.82, SE = .83, *p* = .028) and broadened awareness by time 4 (B = 1.75, SE = .92, *p* = .049) than CBT. No other between-groups differences were observed.

In support of our hypotheses, change in attentional control by Time 2 (post-treatment) was significantly associated with change in decentering by Time 3 (3 month follow-up). Similarly, change in decentering was significantly associated with change in broadened awareness by Time 4 (6 month follow-up). In turn, change in broadened awareness was significantly associated with change in reappraisal by Time 5 (9 month follow-up). Finally, change in reappraisal was significantly associated with change in positive affect by Time 6 (12 month follow-up). The full model accounted for 42% of the variance in change in positive affect by Time 6.

Because significant between groups differences were observed for decentering and broadened awareness, we tested whether changes in these variables mediated the effect of treatment (MBSR vs. CBT) using the SPSS PROCESS 2.13 macro with bootstrapping procedures. Unstandardized indirect effects were computed for each of 1,000 bootstrapped samples, and the 95% confidence interval was computed by determining the indirect effects at the 2.5th and 97.5th percentiles. Significance of the indirect effect was indicated by the upper and lower limits of the 95% confidence interval not spanning zero. This method has been recommended as superior to a normal theory approach to testing mediation (e.g., Sobel test) because it does not assume normality of the indirect effect sampling distribution [72]. First, we examined the indirect effect of MBSR vs. CBT on increases in broadened awareness (by 6 month follow-up) via increases in decentering (by 3 month follow-up). The indirect effect was significant, B = 1.20, SE = .47 (95% CI: .38, 2.27). Next, we observed a significant indirect effect of MBSR vs. CBT on increases in reappraisal (by 9 month follow-up) via increases in broadened awareness (by 6 month follow-up), B = .16, SE = .09 (95% CI: .01, .40). Taken together, these findings indicate that mindfulness-training related increases in decentering mediate the effect of mindfulness training on broadening awareness, which in turn mediates enhanced reappraisal efficacy.

Finally, a series of 10 alternative model configurations were examined, replacing each variable at each time point to consider all permutations (Table 2). The proposed model theoretically consistent with the MMT (Model 1) was the only model in which all paths were significant and fit was excellent.

**Table 2:** Fit indices for final multivariate path model (Model 1) and alternative model specifications.

	Model Iteration									*X*^*2*^	*df*	*p*	CFI	TLI	RMSEA
	Posttreatment		3 Months		6 Months		9 Months		12 Months						
1	Attention	→	NonReacting	→	Observing	→	Reappraisal	→	Positive Affect	36.87	31	.22	.97	.93	.04
2	Attention	X	Observing	X	Reappraisal	→	NonReacting	→	Positive Affect	43.60	31	.07	.92	.83	.06
3	Attention	→	Reappraisal	X	Observing	→	NonReacting	X	Positive Affect	49.20	31	.02	.90	.78	.07
4	Attention	→	Reappraisal	→	NonReacting	→	Observing	X	Positive Affect	52.98	31	.008	.88	.75	.08
5	Positive Affect	→	Observing	X	Reappraisal	X	NonReacting	X	Attention	50.53	31	.015	.87	.72	.08
6	Attention	→	NonReacting	X	Reappraisal	X	Observing	X	Positive Affect	54.67	31	.005	.85	.69	.09
7	Positive Affect	→	NonReacting	X	Reappraisal	X	Observing	X	Attention	54.89	31	.005	.82	.62	.09
8	Positive Affect	→	NonReacting	→	Observing	→	Reappraisal	→	Attention	57.73	31	.002	.83	.64	.09
9	Positive Affect	→	Reappraisal	X	Observing	→	NonReacting	X	Attention	58.44	31	.002	.83	.64	.09
10	Positive Affect	→	Observing	→	NonReacting	X	Reappraisal	→	Attention	61.11	31	.001	.82	.62	.10
11	Positive Affect	→	Reappraisal	→	NonReacting	→	Observing	X	Attention	62.59	31	.001	.82	.61	.10

Note: → indicates a significant path between constructs, whereas an X indicates a nonsignificant path between constructs. Constructs were entered into the model as residualized change scores by covarying pre-treatment levels of each variable. All permutations were not possible due to the fact that attentional control and positive affect were only measured at post-treatment and 12-months follow-up time points.

## Discussion

The Mindfulness to Meaning Theory (MMT) specifies a novel model of mindful positive emotion regulation. Findings from the present multivariate reanalysis of longitudinal data from a sample of treatment-seeking participants with social anxiety disorder suggest that the therapeutic mechanisms specified by the MMT are significantly and prospectively associated in the temporal order proposed by the theory. More specifically, findings suggest that the mechanisms of change identified by the MMT–attentional control, decentering, broadened awareness, and reappraisal–form an iterative chain that promotes long-term increases in positive affectivity in this clinical population. Indeed, an increased ability to focus and shift attention by the end of treatment appears to support the tendency to decenter from distressing thoughts and feelings by three months after treatment. In turn, increased decentering capacity by three months posttreatment predicted greater tendencies toward broadened awareness of interoceptive and exteroceptive information. Broadening of awareness by six months post-treatment was associated with growth in reappraisal self-efficacy by nine months after treatment, suggesting that increased access to novel contextual information may fuel adaptive reconstrual of the meaning of adverse life events. Ultimately, increases in reappraisal occasioned increased positive affect by one year after treatment. Mindfulness-based intervention (i.e., MBSR) appears to specifically boost this longitudinal cycle of therapeutic change by producing significantly greater increases in decentering and broadened awareness than CBT that mediated the effect of mindfulness training on downstream processes–providing support for the foundational assumption in the MMT that mindfulness meditation may be a key means of stimulating positive psychological states.

### The MMT and common mechanisms of therapeutic change

Though significant between-groups differences were observed for decentering and broadened awareness, the MMT’s proposed mechanisms of change may reflect common therapeutic mechanisms that cut across mindfulness-based and cognitive-behavioral interventions. Rigorous RCTs comparing mindfulness-based interventions and CBT have failed to show differential treatment effects on a whole range of mechanisms, including dispositional mindfulness, reappraisal, self-efficacy, acceptance, catastrophizing, and positive and negative affect [11,73,74]. To be clear, given the relative paucity of studies that have compared mindfulness to CBT with respect to their mechanisms of action, it is likely that the therapeutic mechanisms differentiating mindfulness from CBT have not yet been measured and identified in a clinical trial (for example, these treatments may differ with regard to the extent to which they induce nondual awareness and other modes of existential awareness, see [43]. However, it is also possible that any form of therapy that enhances state and trait mindfulness might stimulate the cascade of cognitive-affective processes implicated in the MMT.

Goldin et al. [11] contend that the capacity for metacognitive awareness, reflected by enhanced attentional control coupled with a decentered stance towards the objects of attention, may reflect a central change mechanism encouraged by both CBT and MBSR–a contention supported by results from this study. While CBT aims to promote decentering through explicit training in the practice of attending to and disputing negative thoughts through thought records, MBSR promotes decentering through the practice of mindfulness, which involves re-orienting attention to breath and body sensations to disengage from mental and emotional proliferation while attending to such psychological contents from a non-reactive stance. In both cases, the capacity to decenter from distressing thoughts and feelings is grounded in the ability to shift and sustain attention on salient psychological experiences without becoming overwhelmed by emotional distress. Regardless of the therapeutic means employed, decentering appears to be a common mechanism underlying the effects of mindfulness and reappraisal [75]. Nonetheless, in the present study, MBSR led to significantly greater increases in decentering as assessed by the FFMQ non-reactivity subscale than CBT (as well as significantly greater increases in broadened awareness as assessed by the FFMQ observe subscale), suggesting that mindfulness training may yield specific benefits with regard to stimulating the cascade of positive psychological mechanisms specified in the MMT.

Furthermore, similar improvements in participants’ abilities to cognitively reappraise in both the CBT and MBSR groups appears to support the MMT’s claim regarding the close connection between decentering, broadened awareness, and reappraisal. Interestingly, while CBT provides direct instruction in reappraisal, MBSR does not. The organic development of reappraisal capacities in MBSR participants lends further support for the *mindful reappraisal hypothesis* of the MMT, which posits that mindfulness enhances the capacity for reappraisal [27]. Recent, temporally dynamic growth curve modeling complements earlier cross-sectional research [57] by indicating that the trajectory of increases in state mindfulness (i.e., decentering) over the course of a mindfulness-based intervention is positively associated with increases in reappraisal frequency over that same time period (See S1 File Footnote 1 for more detail) [64]. Present study findings expand upon this observation by implicating the role of broadened awareness to contextual information as a mediator of the decentering-reappraisal relation. In MBSR, this broadening may be the result of cultivating awareness of interoceptive and exteroceptive sensations and perceptions in the context of formal and informal meditation, practices which may counter biased information processing due to the attentional narrowing that occurs in response to negative emotions [76]. Comparatively, in CBT the empirical identification of confirmatory and disconfirmatory evidence for maladaptive beliefs and cognitions may be one method by which the cognitive-behavioral approach promotes broadened awareness of internal and external context and thereby facilitates reappraisal.

### Implications for treatment development

The attentional and cognitive capacities examined in the present study were found to support greater increases in positive affect over the course of a year–providing support for the notion that positive cognitive-emotional states interact to produce durable improvements psychological well-being [76]. Insofar as both CBT and MBSR have been shown to ameliorate psychological distress and target the aforementioned mechanisms of attentional and cognitive regulation (albeit through distinct therapeutic techniques), it may be that psychological interventions designed to explicitly address both the attentional, metacognitive training foundational to MBSR, as well as the cognitive reappraisal training explicated in CBT would be even more efficacious than either of these therapeutic approaches in isolation.

In that regard, Mindfulness Oriented Recovery Enhancement (MORE) is a recently developed mindfulness-based intervention informed by the MMT that integrates traditional mindfulness meditation techniques with explicit cognitive reappraisal training [77,78]. A recent randomized controlled trial (N = 180) provided preliminary evidence for the comparative superiority of MORE to CBT [79], with participation in MORE associated with significantly greater improvements in affect and craving among a sample of inpatients with a variety of co-occurring psychiatric and substance use disorders. Within a behavioral medicine context, MORE has been shown to produce clinically significant improvements in chronic pain symptoms and prescription opioid misuse [80]. However, whether interventions like MORE which combine explicit training in mindfulness and reappraisal promote stronger coupling between constructs specified in the MMT or lead to better treatment outcomes than CBT or MBSR are empirical questions that should be explored by future randomized controlled trials.

### Summary and limitations

Despite the results from this study resonating with previous theoretical and empirical work, limitations should also be noted. First, our ability to model the MMT was constrained by the measures collected during this clinical trial. This was a post-hoc secondary data analysis that attempted to fit clinical trial data to the MMT, and as such, we were limited to the variables that were available in the dataset. In that regard, no direct measure of decentering was available, and so we employed the non-reactivity subscale of the FFMQ to assess this construct. Such use is justified because of the face validity of the items with respect to the construct of decentering, as well as previous findings indicating decentering and the non-reactivity subscale are highly interrelated, N = 461, r = .74 [81], potentially measuring a common, underlying construct. However, it should be noted that decentering and non-reactivity are not identical constructs. Decentering refers to the ability to view one’s experience as mental events as opposed to representations of reality, whereas non-reactivity refers to the ability to remain equanimous in the face of distressing thoughts and feelings, and ability that can ostensibly be achieved through decentering. In that regard, theorizing by Bernstein et al. suggests that decentering itself is comprised of metacognitive awareness, disidentification, and reduced reactivity [40]. The FFMQ non-reactivity subscale includes items pertaining to at least two of these processes: disidentification (e.g., “I watch my feelings without getting lost in them”) and reduced reactivity (e.g., “When I have distressing thoughts or images I am able just to notice them without reaction”). On the other hand, it should be noted that recent factor analytic research did not fully support Bernstein and colleagues’ three proposed metacognitive processes of decentering [82]. Also, in this particular study, the FFMQ non-reactivity subscale was classified as a measure of “intentional non-reactive meta-awareness” and shown to be significantly but modestly correlated with the ability to intentionally adopt a decentered perspective (82), suggesting that the non-reactivity subscale may partially capture key elements of decentering but not completely reflect the construct. Though vigorous debate continues around the operationalization and measurement of the decentering, the present study was limited in its reliance on the FFMQ non-reactivity subscale, which may not provide a nuanced and full representation of this construct.

Similarly, the FFMQ observe facet is more circumscribed in scope than the MMT concept of broadened awareness, which pertains to attention to wide a range of contextual data from which reappraisals can be generated. In contrast, the FFMQ observe facet specifies awareness of a limited set of sensorial and perceptual experiences. Further, complete modeling of the MMT was impossible due to the dataset’s lack of a measure of meaning in life, which is conceptualized as the distal output of the proposed mindfulness-to-meaning process [26]. Future investigations should ensure the robustness of the MMT by using other measures of the proposed constructs, including neurocognitive tasks and psychophysiological assays.

Second, generalizability of these results are limited due to this sample being constituted by individuals with social anxiety disorder. It may be that the relationships observed in this study will differ across individuals with other types of psychological disorders. Continued examination of the MMT in a range of clinical and non-clinical samples is encouraged. Also, this study used a modified, 12-week version of MBSR to match the CBT intervention in terms of time-in-treatment. It is not known how the MMT would map onto a standard length version of MBSR.

Lastly, the temporal dynamics of the MMT are far from established; it is not yet known on what timescale (e.g., seconds, minutes, hours, days, or longer) the process of mindful positive emotion regulation unfolds. The time points studied in this investigation were predetermined by exigencies in the parent clinical trial from which the present data are derived. In that regard, though research by Fredrickson and colleagues suggests that positive affect may broaden the scope of attention [83], the current analysis is unable to ascertain whether inclusion of positive affect and attention at the other time points might have resulted in better fit indices compared to the current established model. It is likely that the MMT might be expressed differently within a given emotion regulatory episode as compared to its expression across numerous emotion regulatory episodes (such as what might occur across the 12 months of data collection in the present study). Moreover, though our aim was to model the core constructs of the MMT with the available data, it is possible that other model specifications with fewer variables or different variables might have fit the data better. That said, the proposed model was the best fitting of 10 alternative models tested. In addition, the temporally dynamic change process may have differed in within-group analyses, which are empirically justified when the null hypothesis of measurement invariance is rejected (See S1 File Footnote 2 for more detail). For multi-group path modeling, >100 cases/observations per group are required [84]. In that regard, in disaggregated multivariate path modeling efforts, the analysis was underpowered and our MMT model did not fit the observed data well when the treatment arms were analyzed separately (See S1 File Footnote 3 for more detail). Thus, in the present dataset the MMT model only fit the observed data when the sample was analyzed as a whole with treatment group modeled as an exogenous variable.

The MMT proposes that linkages between mindfulness and reappraisal emerge at multiple levels of temporal resolution, in keeping with iterative process models of emotional experience [85] and extended process models of emotion regulation [86]. In that regard, the MMT asserts that in the immediate wake of a stressor, attentional control and decentering attenuate negative attentional biases and maladaptive elaborative habits, allowing reappraisal to enter into the iterative emotion regulatory process to modulate the impact of a negative event. Over more extended periods of time, recurrently cultivating metacognitive awareness enables reflective processes to magnify the affective benefits of reappraisal and generate eudaimonic well-being. It is possible that flexible positive emotion regulation requires initial momentary disengagement from elaborative self-referential processing through mindfulness as a precursor to more temporally-extended metacognitive reflection on the self-in-context when hedonic goals must be balanced by eudaimonic values [87]. In that regard, new contemplative science models suggest that metacognitive self-regulation through mindfulness may facilitate fluid reconstrual of the implicational meaning of one’s self-concept and view of reality via decentering and more advanced modes of existential awareness in which conceptual representations of self and world become de-reified [43].

To unpack these questions, more research is needed to explore the MMT at different levels of temporal resolution. Further, the MMT should be modeled through studies designed in an *a priori* fashion to test the hypotheses integral to this theory. For instance, lab-based mindfulness inductions could be used to test the effects of mindfulness mediation on boosting performance-based measures of reappraisal via attentional control, decentering and broadening of awareness, the latter of which might be measured with cognitive assays like the global-local task [88]. Or, ecological momentary assessments could be used as in recent studies [89] to examine time-lagged relations between moment-to-moment changes in attention, decentering, broadened awareness, reappraisal, and positive affect.

The present study should be considered heuristic rather than confirmatory because this was a post-hoc secondary data analysis modeling the MMT with existing data not originally collected for this purpose. Moreover, it is not yet known whether these findings can be generalized beyond social anxiety disorder. Nonetheless, this study makes several novel contributions to psychological science, including providing the first longitudinal test of proposed linkages between core MMT components, as well as expanding understanding of core change mechanisms operating during both CBT and MBSR. Though formal mindfulness meditation appears to uniquely stimulate the mindful positive emotion regulation process by boosting decentering and broadened awareness to a greater extent than CBT, from a transtherapeutic perspective [90], durable positive affectivity may arise from reappraising the meaning of daily adversity, a second-order valuation process [86] fueled by increased apperception of what is beautiful, life affirming, or good in life–an awareness made possible by cultivating attentional capacity in service of decentering from the varieties of mental suffering.

## Supporting information

S1 File Footnotes. (DOCX)
