# Supplementary material for: Testing the mindfulness-to-meaning theory: Evidence for mindful positive emotion regulation from a reanalysis of longitudinal data
Source: PLoS One. 2017 Dec 6;12(12):e0187727. doi: 10.1371/journal.pone.0187727 (PMC5718463; doi:10.1371/journal.pone.0187727)
Supplement: S1 File — (DOCX) [file pone.0187727.s001.docx]

**Footnote 1.** In response to a reviewer comment, to test the “mindful reappraisal” hypothesis of the MMT, we conducted a multivariate autoregressive latent trajectory (m-ALT) model to examine covariation between trait mindfulness (as measured by the total FFMQ score) and reappraisal across all study time points prior to the positive affect outcome at 12 months follow-up. In this model, there was no significant difference between treatment groups on the trajectories of trait mindfulness and reappraisal across the study (treatment group on reappraisal, B = .06, SE = .05, p = .24; treatment group on trait mindfulness, B = -.46, SE = .76, p = .55). However, significant cross-lagged effects indicated that trait mindfulness at each time point predicted reappraisal at the subsequent time point (B = .02, SE = .007, p = .003). There were no significant cross-lagged effects between reappraisal and mindfulness at subsequent time points (B = .36, SE = 1.07, p = .73). There were significant autoregressive effects between each successive time point for trait mindfulness (B = .97, SE = .09, p < .001) and reappraisal (B = .66, SE = .11, p < .001). This m-ALT model fit the data well, χ^2^ = 40.19, df = 32, p = .15, CFI = .98, RMSEA = .05 (95% CI: .00, .09). This m-ALT model provides partial support for the mindful reappraisal hypothesis by indicating that degree of trait mindfulness predicts subsequent degree of reappraisal (irrespective of how trait mindfulness is engendered, by MBSR or CBT).

**Footnote 2.** Multiple group path analysis is empirically warranted when a chi-square difference test of model fit indicates that the proposed model exhibits a significantly different model fit between groups (84) – ruling out the null hypothesis of model invariance. We examined the chi-square difference test (delta χ^2^= 4.49, delta df = 7) which yielded a nonsignificant p-value (p = .72), indicating that model fit did not significantly differ between groups, and therefore the aggregated model was most parsimonious and should be retained as the final model. However, other metrics for determining model invariance have been advanced. Following Cheung & Rensvold (85) a delta CFI of .01 or less indicates that the null hypothesis of invariance should not be rejected. In our comparative analyses of aggregated versus disaggregated models, the CFI of the aggregated model = .90 whereas the CFI of the disaggregated model is = .91, yielding a delta CFI of .01 and suggesting measurement invariance (the aggregated model is most parsimonious). However, it should be noted that Meade, Johnson, & Braddy (86) suggest the superiority of a delta CFI cut-off of .002, which the current delta CFI does exceed.

**Footnote 3.** Despite these power caveats and indications of model invariance, based on a reviewer suggestion we disaggregated the sample and analyzed the model for each treatment group separately. Model fit for the CBT group was poor, exceeding cut-points for adequate model fit: χ^2^ *p-*value = .01, CFI = .80, RMSEA = .12 (.06, .18). Fit for the MBSR group was suboptimal as well: χ^2^ *p-*value = .07, CFI = .88, RMSEA = .09 (.00, .14).
